# Case study of previously healthy 52 years old woman after COVID – 19 infections

**DOI:** 10.1192/j.eurpsy.2025.1272

**Published:** 2025-08-26

**Authors:** I. Ljutica, Z. B. Otasevic, D. Otasevic

**Affiliations:** 1 Psychiatric Clinic, Clinical Center of Montenegro; 2 Dusa, psychiatric branch, Filipovic outpatients clinic, Pogorica, Montenegro

## Abstract

**Introduction:**

Stressful events are important causes of numerous psychiatric disorders, including anxiety disorders, depression and other psychiatric disorders.

The COVID-19 pandemic affects individuals and then all segments of society, and has threatened both physical and mental health.

We have noticed increasing number of the new patients seeking psychiatric help in period after COVID - 19 infections.

Also, tremendously increasing number of sick days.

**Objectives:**

In this case study, we described a 52-year-old female patient. She is a psychologist, an employee in High school in Podgorica working with students. She has achieved 25 years of work experience.She is divorced, has one child. She has never asked for psychiatric help before Sars Covid-19 infection.

She faced first symptoms in terms of bad mood, withdrawal from social contacts, fear, insomnia, problems with attention and concentration. Those symptoms appeared after COVID -19 infections. The patient was hospitalized at the Psychiatry Clinic due to persistent symptoms Admission was due to organic personality changes of the anxious-depressive type, which occasionally reach the level of psychosis. She was absent from the work more than a year.

**Methods:**

Case study

**Results:**

During hospitalization, the patient was treated with anxiolytics, antidepressants and small dosses of antipsychotics medicine.

Her general condition improved after month spent in hospital. She received supportive psychotherapeutic support on a daily basis. She participated in occupational therapy.

A CT scan of the endocranium was normal.

The patient still taking prescribed therapy and regularly attends psychiatrics checks - ups.

She went back to a work after two months.

**Conclusions:**

Based on our daily work with patients, we came to the conclusion that sars covid-19 infection has led to a significant increase in mental health symptoms among the population in Montenegro. This problem must not be ignored”You don’t need to fear anything in life, you just need to understand each other.” Now is the time to understand more so that we would fear less” – Marie Currie

**Disclosure of Interest:**

None Declared

